# Quality of Online Patient Information on Surgical Management of Hidradenitis Suppurativa: A Comprehensive Assessment Using the mEQIP Tool

**DOI:** 10.3390/jcm14227990

**Published:** 2025-11-11

**Authors:** Marco Marcasciano, Martina Astolfi, Medea Pintaudi, Emanuele Vittori, Giuseppe Antonio D’Amico, Alessia Pagnotta, Luigi Bennardo, Michele Rosario Colonna, Steven Paul Nisticò, Manfredi Greco

**Affiliations:** 1 Department of Plastic and Reconstructive Surgery, University “Magna Græcia” of Catanzaro, 88100 Catanzaro, Italy; 2 Department of Plastic and Reconstructive Surgery, Azienda Ospedaliera “Gaetano Martino” of Messina, University of Messina, 98124 Messina, Italy; 3 Hand and Microsurgery Unit, Jewish Hospital, 00186 Rome, Italy; 4 Science of Health Department, School of Medicine, University Magna Graecia, 88100 Catanzaro, Italy; 5 Department of Dermatology, Policlinico Umberto I, Sapienza University of Rome, 00161 Rome, Italy; 6 Plastic and Reconstructive Surgery Unit, “Santa Maria Goretti” Hospital, Sapienza University of Rome, 04100 Latina, Italy

**Keywords:** hidradenitis suppurativa surgery, hidradenitis suppurativa surgical treatment, hidradenitis suppurativa excision, hidradenitis suppurativa surgical management, hidradenitis suppurativa reconstructive techniques

## Abstract

**Background:** Hidradenitis suppurativa (HS) is a chronic inflammatory disorder characterized by recurrent nodules, abscesses, and sinus tracts in apocrine gland-bearing areas. Surgery plays a key role in moderate-to-severe disease. As patients increasingly rely on the internet for decision-making, the quality of online information on HS surgery requires critical evaluation. Previous studies have shown poor quality and limited coverage of surgical aspects. This study systematically assesses publicly available websites on the surgical and reconstructive management of HS, quantifies their quality using the modified Ensuring Quality Information for Patients (mEQIP) tool, and identifies areas needing improvement to support informed decisions. **Methods:** Google, Bing, and Yahoo were searched using five HS surgery-related keywords. The first 50 results per keyword and engine were collected (n = 750), and 214 websites met the inclusion criteria. Sites were categorized by provenance (practitioners, hospitals, healthcare portals, professional societies, encyclopedias) and assessed using the 36-item mEQIP checklist. High quality was defined as ≥23/36 (75th percentile). Comparisons were made by publication era (pre-/post-COVID-19) and source type. **Results:** The mean mEQIP score was 21.7; only 51 websites (23.8%) met the high-quality threshold. No significant difference emerged between pre- and post-COVID publications. Healthcare portals scored highest (22.8), followed by practitioners (21.5) and hospital sites (21.2); professional societies (19.7) and encyclopedias (17.3) performed worst. Major deficiencies included limited discussion of surgical risks, quality-of-life outcomes, and postoperative care. **Conclusions:** Online resources on HS surgery are frequently incomplete and omit essential details on risks, recurrence, and reconstructive options. Surgeons should direct patients toward vetted sources, and professional societies should develop accessible, evidence-based patient guidelines.

## 1. Introduction

Hidradenitis suppurativa (HS) is a chronic inflammatory disorder [1] of the pilosebaceous unit that affects apocrine-gland areas such as the axillae, groin and inframammary folds. It manifests as recurrent nodules, abscesses and sinus tracts that cause pain, scarring and social stigma. Estimates suggest it affects up to 4% of the population, with a higher prevalence among women [2]. The chronic inflammation associated with HS can, in rare but significant cases, lead to malignant transformation, most commonly to squamous cell carcinoma, highlighting the importance of effective long-term disease management [3]. When medical therapy fails or fibrosis and sinus formation are present, surgery becomes a cornerstone of handling this issue [4,5].

The surgical approach of HS is diverse and often challenging. For early, localized disease, deroofing or unroofing may suffice; this involves excision of the roof of sinus tracts to convert them into open wounds that can heal by secondary intention. Limited excisions target discrete nodules or sinus tracts without removing surrounding tissue. Wide excision extends into healthy margins and may be left to heal by secondary intention or be reconstructed with split-thickness skin grafts or flap techniques [6,7]. In advanced disease with extensive scarring or multiple tracts (Hurley stage III), radical excision of the entire affected area is sometimes necessary, followed by reconstruction using locoregional, perforator or free flaps. Each technique carries its own risk-benefit profile: deroofing has low recurrence but may require multiple sessions [8]; wide excision offers durable disease control but results in larger wounds and potentially conspicuous scars; flap reconstruction can restore function and provide better cosmetic outcomes but demands surgical expertise [9]. Recent innovations in wound closure aim to improve outcomes further; for instance, the co-grafting of acellular dermal matrix (ADM) with split-thickness skin grafts (STSG) has shown promise in accelerating wound closure and improving scar elasticity [10]. Postoperative care involves wound management, physiotherapy, analgesia and prevention of recurrence [11].

With the growing number of surgical techniques available, many patients now search online before meeting a surgeon, often arriving with expectations shaped by variable-quality information [12]. Surveys indicate that more than two-thirds of patients search online for health information [13]. Previous research has shown that websites describing surgical procedures frequently omit critical information on risks, outcomes and alternatives, and may lack transparency about authorship and evidence sources.

A previous study on general HS information by Karamitros et al. found major gaps in completeness and reliability [14]. Nevertheless, to our knowledge, no report has specifically addressed patient-facing information on HS surgical reconstructive options, nor contextualized it within the pre-COVID landscape and the post-COVID acceleration of digitalization, which may have influenced its evolution. Building on these premises, the present study expands current understanding of HS-related online information by focusing on the specific domain of surgical and reconstructive management.

Through the application of the validated modified Ensuring Quality Information for Patients (mEQIP) tool, it offers a structured and comprehensive assessment of information quality, while the inclusion of a temporal comparison between pre- and post-COVID-19 periods provides additional insights into how digital health communication in this area has evolved over time.

By analyzing scores across website categories and timeframes, we systematically evaluated publicly available resources to identify current strengths and gaps and to propose evidence-based directions for improvement.

## 2. Materials and Methods

### 2.1. Search Strategy

This cross-sectional study was designed and reported following the principles of the STROBE (Strengthening the Reporting of Observational Studies in Epidemiology) statement where applicable. A completed STROBE checklist can be provided as Supplementary Materials [15]. We conducted a systematic search using three leading search engines (Google^®^, Yahoo!^®^, and Bing^®^) to obtain a representative dataset of publicly accessible online content regarding the surgical management of Hidradenitis Suppurativa (HS) [16]. We selected five high-volume and clinically relevant English-language keywords, identified via Google Trends and medical SEO analytics: “hidradenitis suppurativa surgery”; “hidradenitis suppurativa surgical treatment”; “hidradenitis suppurativa excision”; “hidradenitis suppurativa surgical management”; “hidradenitis suppurativa reconstructive techniques.” These terms were intended to capture a broad range of surgical information sought by patients. For each keyword, the first 50 results from each search engine were recorded to approximate typical patient behavior, yielding 750 potential URLs. Searches were performed in a fresh browser session with cache and cookies cleared and geolocation fixed to New York, USA, to minimize personalization bias. All results were exported into a spreadsheet for screening (Table 1).

### 2.2. Screening and Eligibility Criteria

Initial screening and deduplication were conducted by two authors (M.A., M.P.). Duplicate links, broken links, pages behind paywalls and non-informative content (e.g., videos without transcripts, image galleries without explanatory text, or user-generated forum posts) were excluded. Websites not focusing on surgical topics—such as those discussing only medical therapy or lifestyle management—and commercial advertisements that lacked substantive educational content were eliminated. Pages were included if they contained textual information aimed at patients about surgical management of HS and were freely accessible. Importantly, we did not limit inclusion to English; thus, non-English websites were translated using a professional-grade automated tool to allow consistent evaluation. After initial screening and deduplication, 214 unique websites met our inclusion criteria and were selected for full analysis. Any disagreements between the two initial reviewers (M.A. and M.P.) regarding study inclusion were discussed and resolved through consultation with a third investigator (E.V.) until consensus was achieved.

### 2.3. Classification and Scoring

Each website was categorized by provenance into one of five groups: individual practitioners or private clinics; hospitals and medical centers; general healthcare portals (commercial or general medical information sites); professional societies (including medical associations or governmental agencies); and encyclopedic sources, such as Wikipedia. Two reviewers (M.A., M.P.) independently assigned categories; disagreements were resolved through discussion until consensus was reached with the aim of a third investigator (E.V.). Quality assessment was conducted with the modified Ensuring Quality Information for Patients (mEQIP) tool. This checklist comprises 36 binary items organized into three domains. The content domain (items 1–18) examines whether the site defines HS, explains its causes and treatment options (both surgical and nonsurgical), outlines the benefits and risks of surgery, addresses postoperative management, and discusses quality-of-life outcomes and cost considerations. The identification domain (items 19–24) assesses transparency through disclosure of authorship, institutional affiliations, sources of funding, publication or update dates and references to supporting literature. The structure domain (items 25–36) evaluates readability (plain language, avoidance of jargon, short sentences), organization of information, balance between benefits and risks, use of visuals (diagrams, videos or infographics), design and accessibility features (such as print-ready forms or space for notes) (Table 2). Each item was scored as present (1) or absent (0) [17]. To ensure our quality assessment was objective and grounded in existing research, we adopted a methodology consistent with prior EQIP-based studies [18,19]. Accordingly, we defined ‘high-quality’ websites as those scoring at or above the 75th percentile of our sample’s distribution. This approach uses a relative, data-driven cutoff rather than an arbitrary absolute score, thereby providing an established and reproducible benchmark for our quality threshold. In our dataset, this threshold corresponded to 23 points. Inter-rater reliability was calculated using Cohen’s kappa statistic. Statistical analyses were performed using SPSS version 29 for macOS. Differences between continuous variables were assessed using Student’s *t*-test or one-way analysis of variance (ANOVA), while categorical variables were compared with χ^2^ or Fisher’s exact tests. Given that the mEQIP total score is an ordinal-like summary measure, its use with parametric tests such as ANOVA is considered robust with a sufficient sample size and is a common practice in similar research evaluating quality scoring tools. Statistical significance was set at *p* < 0.05.

## 3. Results

### 3.1. Website Characteristics and Scores

**Figure 1:**
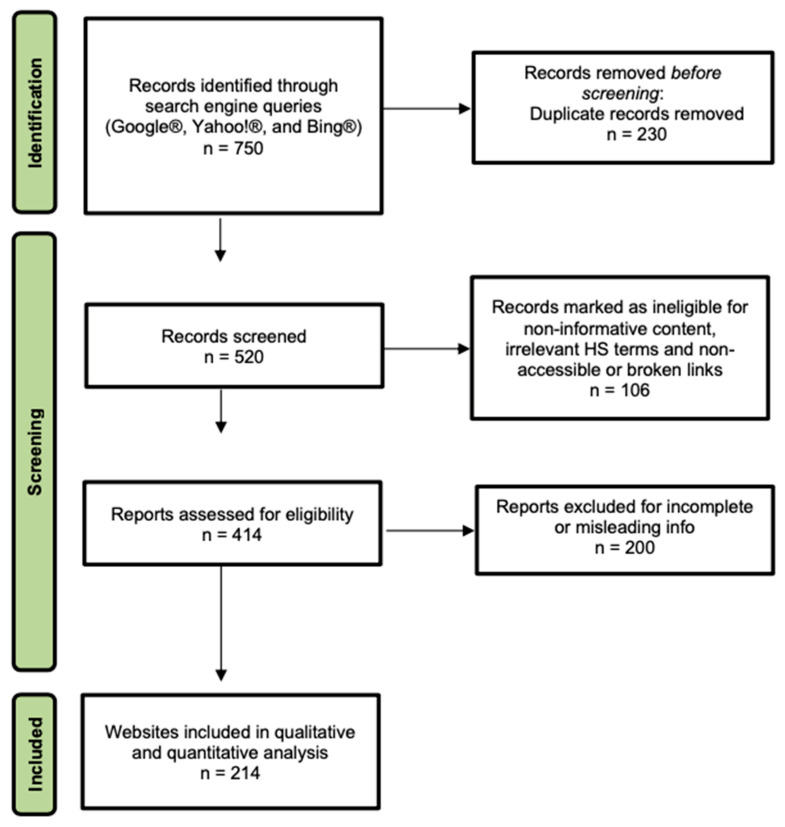
PRISMA Flow Diagram for Website Identification and Selection. The figure details the four-stage process used to select publicly available websites for quality analysis. Records initially retrieved from Google^®^, Yahoo!^®^, and Bing^®^ (Identification) were screened for duplicates and relevance (Screening). Full pages were then assessed for eligibility based on their specific focus on the surgical and reconstructive management of Hidradenitis Suppurativa (Eligibility), leading to the final cohort of 214 websites included in the study (Inclusion).

The flow of search, screening and selection is summarized in Figure 1. After removing duplicates and excluding irrelevant or inaccessible pages, 214 unique websites were included in the analysis. Approximately 46.7% of these websites were affiliated with hospitals or medical centers (n = 100). General healthcare portals comprised 30.4% (n = 65). Individual practitioners or private clinics accounted for 15.4% (n = 33). Professional societies, such as dermatologic or surgical associations, represented 5.1% (n = 11). Encyclopedic sources accounted for the smallest fraction, at 2.3% (n = 5). This distribution reflects the diverse sources from which patients may seek information.

### 3.2. mEQIP Scores Across Websites

The evaluation of online patient-facing resources concerning the surgical management of HS was conducted using the mEQIP score, as detailed in Table 3 [17]. Across the entire cohort, the mean mEQIP score was 21.7 with a standard deviation of 4.8 (median = 22, IQR = 20–24). Scores ranged from 9 to 32. Using the 75th percentile cut-off (23 points), 51 websites (23.8%) were classified as high quality, while 163 (76.2%) fell below that threshold (Table 4). Inter-rater reliability between the two primary reviewers was substantial, with a Cohen’s κ = 0.76, indicating “substantial agreement” based on established benchmarks and underscoring the consistency of our scoring methodology.

When analyzed by source category, general healthcare portals achieved the highest mean score (22.8 ± 4.5). Websites authored by individual practitioners scored a mean of 21.5 ± 4.6, and hospital websites averaged 21.2 ± 4.8. Professional society pages had a lower mean score (19.7 ± 5.1), and encyclopedic sources scored worst with a mean of 17.3 ± 3.9. Differences in mean mEQIP scores among categories were statistically significant (*p* < 0.05) according to the one-way ANOVA test. Notably, all encyclopedic sites and 86% of professional society websites were classified as low quality, highlighting substantial room for improvement even among authoritative organizations.

### 3.3. Domain-Specific Performance

#### 3.3.1. Surgical and Risk Communication

A large majority of websites (82%) provided a basic definition of HS and acknowledged surgery as a treatment option for advanced diseases. However, only 36% discussed medical or non-surgical alternatives, and just over one third (38%) explained how surgical techniques differ depending on disease stage or anatomical location. A small proportion (28%) elaborated on the potential benefits of reconstructive surgery, such as pain relief, improved mobility or enhanced quality of life. Notably, qualitative descriptions of risks—such as infection, wound dehiscence, flap necrosis or neuropathic pain—were found in only 3.7% of websites, and quantitative risk data (e.g., estimated rates of recurrence or complications) were provided by just 3.3%. Postoperative management guidance appeared on only 12% of websites, while 39% mentioned strategies for managing complications should they occur. Less than one-quarter of sites (22%) addressed the financial implications of surgery or insurance coverage. Discussion of psychosocial impact and quality of life following surgery was present in 37% of websites. Overall, the content domain underscores a major deficit in balanced, comprehensive education for patients.

#### 3.3.2. Transparency and Credibility

In the identification domain, transparency was variable. Just over half (54%) of websites disclosed authorship or institutional affiliation, and only 29% displayed a publication or update date, leaving readers unsure of the currency of information. References to supporting evidence were included in 28% of sites. Only 11% provided information on funding sources or sponsorship, and a mere 5% acknowledged any form of patient involvement in developing the content. These findings demonstrate persistent gaps in accountability and reliability.

#### 3.3.3. Structural Quality

The structural quality of websites fared better. All websites (100%) used plain language and explained technical terms. Short sentences were employed in 93% of sites, and information was organized logically in 68%. A balanced presentation of the risks and benefits of surgery was present in 60% of pages. Adequate design and layout—characterized by clear headings, readable fonts and appropriate spacing—were observed in 75% of websites. However, only 40% of sites used diagrams, photographs or videos to illustrate surgical procedures, wound care or anatomy, limiting visual comprehension. Features to facilitate patient engagement, such as space to record questions or printable consent forms, were scarce (32% and 12%, respectively). Overall, this study highlights that while readability is generally acceptable, interactive and visual elements that could enhance understanding remain underutilized.

### 3.4. Temporal Trends

The COVID-19 pandemic acted as an unprecedented catalyst for digital health adoption, fundamentally altering how patients access medical information. With significant reductions in in-person healthcare utilization globally due to lockdowns and fear of infection [20], patients were increasingly compelled to turn to online resources to understand complex conditions like HS and their surgical options. This shift created a critical need—and a unique opportunity—for the medical community to enhance the quality and reliability of digital patient education, as individuals strongly relied upon the Internet to stay informed [21]. We hypothesized that this surge in demand would correspond with an improvement in the quality of newly published online materials.

However, our analysis reveals a concerning stagnation. To investigate this, we compared websites with identifiable publication dates, categorizing them as pre- or post-pandemic (using the WHO’s declaration date of 11 March 2020, as the cutoff) [22]. Of the dated websites, 87 were published post-pandemic and 76 pre-pandemic. The mean mEQIP scores for these two groups were nearly identical: 21.9 ± 4.7 for post-pandemic sites versus 21.8 ± 4.9 for pre-pandemic sites, a difference that was not statistically significant (*p* = 0.84). This finding suggests that the quality of information did not evolve to meet the heightened needs of patients during this period (Table 5) [18,19].

Furthermore, websites lacking a publication date (n = 51)—which are often older or less rigorously maintained—had a significantly lower mean score of 19.0 ± 5.2 compared to dated sites (*p* < 0.05). This underscores that transparency via dating correlates with higher quality and that a substantial portion of accessible information is likely outdated. Ultimately, our data point to a significant missed opportunity. Despite a paradigm shift that placed more responsibility on patients to seek information independently, the quality of online resources on HS surgery failed to improve, leaving a critical gap in patient support when it was needed most.

### 3.5. Additional Analyses

We examined whether website quality varied by language or hosting model. English remained the dominant language across our sample, with a minority of sites in Spanish, French and other languages. Non-English websites scored similarly to English pages, suggesting that language was not a major determinant of quality. Differences between privately hosted sites (practitioner and hospital) and commercial portals were minimal; although practitioner pages sometimes included personal testimonials and portals tended to be more general, their overall mEQIP scores were comparable.

## 4. Discussion

### 4.1. Integration with Current Guidelines

Our study demonstrates that although numerous websites describe surgical options for HS, very few provide comprehensive, evidence-based information. This gap is particularly troubling in light of contemporary treatment guidelines that emphasize a multidisciplinary approach [4,5,6,7,8,9,10,11,12,13,14,15,16,17,18,19,20,21,22,23,24]. Expert consensus recommends tailoring surgical strategy to disease severity and anatomic location, integrating medical and surgical therapy, and continuing biologic agents through the perioperative period to reduce inflammation and improve outcomes [25,26,27]. Wide excision remains the gold standard for advanced disease due to lower recurrence rates compared with deroofing or limited excision [28]. Nevertheless, surgery should be integrated early when reversible inflammatory components exist to spare more extensive procedures. Postoperative maintenance therapy—including lifestyle modifications and targeted biologics—is critical to minimize recurrence [28]. Despite these recommendations, our evaluation found that only 38% of websites detailed how surgical approaches vary across stages of HS, and a mere 12% discussed postoperative management. The absence of this information denies patients a complete understanding of their options, potentially leading to unrealistic expectations, poor adherence to postoperative care, and ultimately, suboptimal surgical outcomes. It is incumbent upon professional societies and health institutions to ensure that online resources reflect current best practices, including the integration of surgery and reconstructive alternatives with systemic therapy [29,30]. The unexpectedly low scores from professional society websites may reflect a primary focus on professional rather than patient audiences, leading to the use of academic language barriers, or less frequent content update cycles compared to commercial health portals.

### 4.2. Aesthetic Outcomes and Quality of Life

Aesthetic outcomes are pivotal for patient satisfaction. Modern reconstructive techniques, such as V-Y advancement flaps, thoracodorsal artery perforator flaps, propeller flaps and perforator-based fasciocutaneous flaps, have transformed HS surgery by allowing radical excision with simultaneous closure, preserving form and function. Studies have shown that flap reconstructions yield lower recurrence rates and better cosmetic outcomes than healing by secondary intention or skin grafts [28,29]. In a prospective cohort of nearly 200 clinic-based HS surgeries, the majority of patients reported that they would undergo surgery again, yet only 58% were satisfied with the appearance of their scars, suggesting room for improvement [31]. Satisfaction was higher among older individuals and those with obesity, whereas smokers and patients who underwent simple excision without closure were less satisfied. This highlights the need for standardized, evidence-based surgical techniques that minimize scarring and align with patient preferences [32].

Innovative technologies are also emerging to address cosmetic concerns. Energy-based devices, such as the Endolift laser—a 1470 nm intralesional diode laser—have been investigated for various dermatological conditions, including HS [33]. Preliminary evidence indicates that this minimally invasive procedure can improve the appearance of scars and attenuate sinus tracts with minimal downtime and mild adverse events [34]. Furthermore, adjunctive therapies like platelet-rich plasma (PRP) are being explored to enhance wound healing after flap surgery, with some studies suggesting a trend towards reduced complications [35]. Although high-quality trials are still warranted, the potential to improve aesthetic outcomes should be considered alongside conventional surgical options to support informed decision-making and realistic patient expectations.

### 4.3. Comparison with Related Literature

Our findings expand on those of Karamitros et al., who analyzed general HS patient information and identified a lack of risk-related content, noting that website popularity did not reflect informational quality [14]. Similar studies on patient information for breast augmentation and bariatric surgery have found comparable issues: low citation of evidence, poor discussion of complications and absence of postoperative care instructions [36,37,38,39,40]. These patterns suggest that the problem is not confined to HS but reflects a broader gap in patient-oriented surgical education online.

### 4.4. Implications for Patient Education

Online resources profoundly influence patient perceptions. Surgeons should proactively inquire about the sources patients have consulted and guide them towards accurate information. Guiding patients to reliable websites or offering tailored educational materials can correct misconceptions and improve postoperative satisfaction. Based on our findings, we propose several recommendations to improve digital patient education:

(a) Comprehensive content: Websites should systematically describe all relevant surgical and reconstructive techniques, emphasizing how choices depend on disease stage and anatomical site. Non-surgical alternatives, such as biologic therapy and lifestyle modification, should also be mentioned to contextualize surgery within multimodal care [37].
(b) Patient-centered guidelines: Professional societies should develop patient-centered HS surgery guidelines using clear language and transparent risk–benefit information [38,39].
(c) Balanced risk–benefit discussion: Clear information on complications, pain, healing time, and recurrence should be mandatory, including quantitative data where available. Potential benefits—such as pain relief, improved mobility, enhanced body image, and quality of life—should be communicated with realistic expectations [40,41,42,43].
(d) Postoperative care information: Instructions on wound care, dressings, activity limits, and warning signs should be provided, along with long-term maintenance strategies like smoking cessation, weight control, and ongoing systemic therapy.
(e) Transparency and credibility: Authorship, credentials, and affiliations should be clearly disclosed. Including publication dates ensures information is current, while references add credibility and enable further reading. Any sponsorship or advertising influence must be transparently stated [44,45].
(f) Interactive tools and quality standards: Visual aids like diagrams, videos, and interactive tools can improve patient understanding of HS surgery and wound care, while decision aids may boost engagement. Implementing quality standards, such as HON code certification or HS-specific checklists, can help identify reliable resources [46,47].

## 5. Conclusions

Online information on HS surgery is abundant but often lacks consistency and depth. Among 214 websites, only a quarter met high-quality standards. Almost no sites discussed modern reconstructive techniques or emerging therapies that could enhance functional and cosmetic outcomes, nor did they emphasize the importance of integration with targeted systemic therapy. Developing transparent, evidence-based online resources is essential. In practice, this would mean websites that clearly explain recurrence rates, potential complications, and recovery times—information most patients ask about during consultation. Better online resources could support shared decision-making and help improve patient satisfaction and adherence.

## 6. Limitations and Future Research

This study has some limitations. Search results are influenced by algorithms, location, and browsing history, which may affect reproducibility. Our use of a fixed geographical setting (New York, NY, USA), while methodologically necessary to reduce variability, may limit the generalizability of the findings to other regions. Although we used a standardized approach, our findings might not reflect all patient experiences. The mEQIP tool’s binary scoring may miss content nuance, and excluding videos without transcripts or forums could have omitted relevant sources [45,48,49]. Additionally, non-English websites were translated with an automated tool, and the absence of verification by a bilingual reviewer means that subtle inaccuracies could have been introduced. Website content was assessed at a single time point and may have changed. We also did not quantify readability or evaluate the impact of search ranking on perceived trust, which future studies could explore using tools like natural language processing and by including social media platforms.

## Figures and Tables

**Table 1:** Primary Search Strategy Summary.

Search Engine	Keywords Used	Initial Results Retrieved	Duplicates Removed	Eligible After Screening
Google	5 keywords	250	91	159
Yahoo!	5 keywords	250	77	173
Bing	5 keywords	250	62	188

**Table 2:** Domains and Structure of the mEQIP Tool.

Domain	Item Numbers	Description
Content	1–18	Covers pathology, treatment options, benefits and risks.
Identification Data	19–24	Assesses authorship, institutional source, and date of publication.
Structure	25–36	Evaluates readability, navigation, and format accessibility for patients.

**Table 3:** mEQIP tool applied to the 214 eligible websites.

Question	Yes (%)	No (%)
Content		
1. Initial definition of which subjects will be covered	206 (96.26%)	8 (3.74%)
2. Coverage of the above-defined subjects	205 (95.79%)	9 (4.21%)
3. Description of the medical problem	206 (96.26%)	8 (3.74%)
4. Definition of the purpose of the medical intervention	180 (84.11%)	34 (15.89%)
5. Description of treatment alternatives (including no treatment)	95 (44.39%)	119 (55.61%)
6. Description of the sequence of the medical procedure	88 (41.12%)	126 (58.88%)
7. Description of qualitative benefits	130 (60.75%)	84 (39.25%)
8. Description of quantitative benefits	40 (18.69%)	174 (81.31%)
9. Description of qualitative risks and side effects	8 (3.74%)	206 (96.26%)
10. Description of quantitative risks and side effects	7 (3.27%)	207 (96.73%)
11. Addressing quality of life issues	79 (36.92%)	135 (63.08%)
12. Description of how potential complications will be dealt with	82 (38.32%)	132 (61.68%)
13. Description of precautions that the patient may take	85 (39.72%)	129 (60.28%)
14. Mention of alert signs that the patient may detect	150 (70.09%)	64 (29.91%)
15. Addressing medical intervention cost and insurance issues	20 (9.35%)	194 (90.65%)
16. Specific contact details for hospital services	110 (51.40%)	104 (48.60%)
17. Specific details of other sources of reliable information/support	115 (53.74%)	99 (46.26%)
18. The document covers all relevant issues on the topic	60 (28.04%)	154 (71.96%)
Identification Data		
19. Date of issue or revision	80 (37.38%)	134 (62.62%)
20. Logo of the issuing body	170 (79.44%)	44 (20.56%)
21. Name of persons or entities that produced the document	120 (56.07%)	94 (43.93%)
22. Name of persons or entities that financed the document	18 (8.41%)	196 (91.59%)
23. Short bibliography of evidence-based data used in the document	40 (18.69%)	174 (81.31%)
24. The document states whether and how patients were involved/consulted in its production	10 (4.67%)	204 (95.33%)
Structure		
25. Use of everyday language explains complex words or jargon	214 (100%)	0 (0%)
26. Use of generic names for all medications or products	190 (88.79%)	24 (11.21%)
27. Use of short sentences	200 (93.46%)	14 (6.54%)
28. The document personally addresses the reader	150 (70.09%)	64 (29.91%)
29. The tone is respectful	210 (98.13%)	4 (1.87%)
30. Information is clear	210 (98.13%)	4 (1.87%)
31. Information is balanced between risks and benefits	160 (74.77%)	54 (25.23%)
32. Information is presented in a logical order	190 (88.79%)	24 (11.21%)
33. The design and layout are satisfactory	200 (93.46%)	14 (6.54%)
34. Figures or graphs are clear and relevant	100 (46.73%)	114 (53.27%)
35. The document has a named space for the reader’s notes	10 (4.67%)	204 (95.33%)
36. The document includes a consent form, contrary to recommendations	5 (2.34%)	209 (97.66%)

**Table 4:** Observed Frequencies of High vs. Low EQIP scores by website category.

Category	High Score (≥23)	Low Score (<23)
Healthcare Portals	18	47
Practitioners	7	26
Hospitals	25	75
Professional Societies	1	10
Encyclopedias	0	5

**Table 5:** High-scoring website by date of publishing.

Date of Publishing or Updating	High-Scoring Websites (n, %)	Mean Overall EQIP
Post-COVID-19	28 (54.9%)	21.93
Pre-COVID-19	8 (15.68%)	21.75
No data of publishing or updating	15 (29.41%)	19.04

## Data Availability

All authors declare that the datasets generated during the study are available in the article itself.

## Supplementary Materials

The following supporting information can be downloaded at: https://www.mdpi.com/article/10.3390/jcm14227990/s1, STROBE Statement—checklist of items that should be included in reports of observational studies.
