# Supplementary material for: Quality of Online Patient Information on Surgical Management of Hidradenitis Suppurativa: A Comprehensive Assessment Using the mEQIP Tool
Source: J Clin Med. 2025 Nov 11;14(22):7990. doi: 10.3390/jcm14227990 (PMC12653717; doi:10.3390/jcm14227990)
Supplement: Supplementary file 1 [file jcm-14-07990-s001.zip › jcm-3957445-supplementary.pdf]

STROBE Statement—checklist of items that should be included in reports of observational studies

"Quality of Online Patient Information on Surgical Management of Hidradenitis Suppurativa: A Comprehensive Assessment Using the mEQIP Tool"

	Item No	Recommendation
**Title and abstract**	1	(*a*) **Abstract, Methods Section.** While the title does not state "cross-sectional study," the abstract clearly describes the methodology: *"This study systematically assesses publicly available websites... quantifies their quality... and identifies areas needing improvement."* The study design is explicitly confirmed in the **Methods (Section 2.1)**: *"This cross-sectional study was designed and reported following the principles of the STROBE statement."*
		(*b*) **Abstract.** The abstract is structured and provides a summary of all key sections: **Background** (problem), **Methods** (search strategy, inclusion criteria, mEQIP tool), **Results** (mean score, percentage of high-quality sites, comparison between sources), and **Conclusions** (key takeaway message).
**Introduction**		
Background/rationale	2	**Introduction (Paragraphs 1, 2, 3).** The introduction establishes the clinical context of Hidradenitis Suppurativa (HS), the role of surgery, and the increasing reliance of patients on online health information. It cites previous literature [8-11] to highlight the known problem of variable-quality online content, thus establishing the rationale for the current study.
Objectives	3	**Introduction (Final Paragraph).** The objective is clearly stated: *"we aimed to systematically assess the quality of publicly available websites dealing with the surgical management of HS... we sought to identify strengths and deficits... and offer recommendations for improvement."* A specific hypothesis regarding temporal trends was also tested, as explained in **Results (Section 3.4)**.
**Methods**		
Study design	4	**Methods (Section 2.1).** The first sentence of the methods section now explicitly states the study design: *"This cross-sectional study was designed and reported following the principles of the STROBE statement."*
Setting	5	**Methods (Section 2.1).** The setting is clearly defined as the online environment accessed via three major search engines (Google®, Yahoo!®, Bing®). The data collection timeframe is contextualized by the temporal analysis, comparing websites published before and after March 11, 2020. The fixed geolocation for searches (New York, USA) is also specified to ensure reproducibility.
Participants	6	**Methods (Section 2.2).** The "participants" are websites. Eligibility criteria are detailed, specifying both inclusion criteria (e.g., *"contained textual information aimed at patients about surgical management of HS"*) and exclusion criteria (e.g., *"Duplicate links, broken links, pages behind paywalls and non-informative content"*).
Variables	7	**Methods (Section 2.3).** The primary outcome variable is the total **mEQIP score**. The primary predictor variable is the **website source category** (practitioners, hospitals, etc.). Other variables analyzed include the **publication era** (pre- vs. post-COVID-19) and **language**.
Data sources/	8*	**Methods (Sections 2.1 and 2.3).** Data sources were three search engines using five

measurement		specific keywords. The measurement tool, the **36-item mEQIP tool**, is described in detail, including its three domains (Content, Identification, Structure) and binary scoring system (present=1, absent=0). The inter-rater reliability was measured using Cohen's kappa.
Bias	9	**Methods (Section 2.1).** Efforts to minimize bias are described: *"Searches were performed in a fresh browser session with cache and cookies cleared and geolocation fixed to New York, USA to minimize personalization bias."* Furthermore, a standardized review process with multiple independent reviewers and a method for resolving disagreements was used (**Sections 2.2, 2.3**).
Study size	10	**Methods (Section 2.1).** The study size was determined by a systematic and pragmatic search strategy: *"For each keyword, the first 50 results from each search engine were recorded to approximate typical patient behavior, yielding 750 potential URLs."* This approach is justified by its alignment with how typical users interact with search engines, as supported by literature [12, 13].
Quantitative variables	11	**Methods (Section 2.3).** The primary quantitative variable, the mEQIP score, was summarized using mean, standard deviation, median, and IQR. It was treated as a continuous variable for comparative analyses (t-test, ANOVA), with a justification provided: *"the use of parametric tests... is considered robust with a sufficient sample size."*
Statistical methods	12	**Methods (Section 2.3).** The statistical methods are explicitly listed: *"Student's t-test or one-way analysis of variance (ANOVA), while categorical variables were compared with χ^2 or Fisher's exact tests."* Inter-rater reliability was assessed with **Cohen's kappa**. The significance level was set at $p < 0.05$.

**Results**		
Participants	13*	(a) **Results (Section 3.1) and Figure 1.** The **PRISMA flow diagram (Figure 1)** visually and numerically details the flow of websites through the selection process: 750 records identified, 230 duplicates removed, 520 screened, 106 excluded, leading to 214 websites included in the final analysis.
Descriptive data	14*	(a) **Results (Section 3.1 and 3.2).** Descriptive data for the websites are provided, including the distribution across source categories (e.g., *"46.7 %... were affiliated with hospitals or medical centers"*). The overall descriptive statistics for the primary outcome are also given (mean mEQIP score = 21.7 ± 4.8; median = 22, IQR = 20-24).
Outcome data	15*	**Results (Section 3.2).** The main outcome is reported: *"Across the entire cohort, the mean mEQIP score was 21.7"* and *"51 websites (23.8 %) were classified as high quality."* Domain-specific performance is detailed in **Section 3.3**, providing percentages for each of the 36 mEQIP items.
Main results	16	**Results (Section 3.2).** The main results are presented, comparing mEQIP scores across different website categories. A statistically significant difference was found ($p < 0.05$), with healthcare portals scoring highest (22.8) and encyclopedic sources lowest (17.3). The lack of significant change over time is also a key finding (**Section 3.4**, $p = 0.84$).
Other analyses	17	**Results (Section 3.5).** An additional analysis is briefly reported regarding language and hosting models, noting no significant differences were found. The comparison of dated vs. undated websites
**Discussion**		
Key results	18	**Discussion (Section 4.1).** The first paragraph of the discussion summarizes the central finding: *"Our study demonstrates that although numerous websites describe surgical options for HS, very few provide comprehensive, evidence-based information."* This directly addresses the study's primary objective.
Limitations	19	**Limitations and future research (Section 6).** A dedicated section discusses several limitations, including the influence of search algorithms, the use of a single geolocation limiting generalizability, potential nuances missed by binary scoring, and the use of automated translation for non-English websites.
Interpretation	20	**Discussion (Sections 4.1, 4.2, 4.3, 4.4).** The findings are interpreted in the context of current clinical guidelines [19-22], aesthetic outcomes [24, 26], and related literature on online health information quality [11, 31-34]. The *"Implications for patient education"* section provides a cautious and constructive interpretation, offering actionable recommendations.
Generalisability	21	**Limitations and future research (Section 6).** Generalizability is directly addressed: *"Our use of a fixed geographical setting (New York, USA)... may limit the generalizability of the findings to other regions."*
**Other information**		
Funding	22	*This research received no external funding.*

*Give information separately for cases and controls in case-control studies and, if applicable, for exposed and unexposed groups in cohort and cross-sectional studies.

Note: An Explanation and Elaboration article discusses each checklist item and gives methodological background and published examples of transparent reporting. The STROBE checklist is best used in conjunction with this article (freely available on the Web sites of PLoS Medicine at http://www.plosmedicine.org/, Annals of Internal Medicine at

http://www.annals.org/, and Epidemiology at http://www.epidem.com/). Information on the STROBE Initiative is available at www.strobe-statement.org.
